# Perceived social support on postpartum mental health:  An instrumental variable analysis

**DOI:** 10.1371/journal.pone.0265941

**Published:** 2022-05-05

**Authors:** John Nkwoma Inekwe, Evelyn Lee

**Affiliations:** Macquarie Business School, Macquarie University, North Ryde, New South Wales, Australia; Sam Houston State University, UNITED STATES

## Abstract

The postpartum period is a challenging transition period with almost one in ten mothers experiencing depression after childbirth. Perceived social support is associated with mental health. Yet empirical evidence regarding the causal effects of social support on postpartum mental health remains scarce. In this paper, we used a nationally representative panel data of women to examine causality between perceived social support and postpartum mental health. We used fixed-effect method and included dependent variable lags to account for past mental health condition before birth (i.e., the pre-pregnancy and prenatal periods). The study also used an instrumental variable approach to address endogeneity. We find a declining trend in postpartum mental health between 2002 to 2018. Our study also showed that past mental health (i.e., before childbirth) is positively correlated with postpartum mental health. A universal routine mental health screening for expectant and new mothers should remain a key priority to ensure mental wellbeing for the mothers and their infants.

## Introduction

Postpartum depression is a mood disorder that begins within the first 4 to 6 weeks after childbirth and lasts as long as one year, with its highest intensity within the first six months. To meet diagnostic criteria for depression, symptoms of depression must be continuously present for at least two weeks and interfere with the individual’s everyday functioning [1]. A recent estimate shows one in ten mothers experienced depression during the first postpartum year [2] although low and middle-income countries have reported higher rates due to limited access to healthcare services [3, 4]. In Australia, women living in socially disadvantaged areas are more likely to experience postpartum depression than women living in higher socioeconomic status. This is shown in a study by Edwards and colleagues who found almost one in three Australian women (29.7%) living in the most socioeconomically disadvantaged community had a history of postnatal depression, although other social factors such as marital difficulties, spousal violence, and negative life events might have contributed to the high prevalence in the community [5].

One of the concerns with undiagnosed postpartum depression is the adverse effect on child development and functioning. While postpartum depression may resolve spontaneously within weeks after its onset, approximately 20% of women still have depression after the first year of delivery, 13% after two years, and 40% will relapse either during subsequent pregnancies or on other occasions unrelated to pregnancy [6]. Mothers suffering from depression often display hostility, negligence, lack of attachment with the infant, and less tolerance to their infant’s needs which may affect child developmental including cognitive and language developments and poor gross and fine motor achievements with adverse consequences found to persist to adolescence [7, 8].

In Australia, there have been several policy responses to address maternal mental health. In 2008, Beyond Blue, a national, independent, and non-profit organisation developed a National Action Plan for Perinatal Health that provides a blueprint for improving maternal mental health through an integrated framework of community, primary care, and specialist services [9]. In the same year, the Australian Commonwealth Government Department of Health established the National Perinatal Depression Initiative (NPDI) to provide routine and universal depression screening for all women as part of pregnancy and postnatal care. National clinical guidelines for perinatal depression recommend universal screening for perinatal depression and anxiety and that all women identified with mental health issues should be provided with a comprehensive mental health assessment [10].

Despite the evidence of benefit in women receiving depression screening and assessment, in 2013, due to fiscal concern, the Australian Government did not extend their funding agreement to provide care for women with perinatal depression [11]. While state-based perinatal mental health initiatives such as the New South Wales Supporting Families Early policy and SAFE START clinical practice guideline are introduced to provide services to identify and treat women who are at risk of depression, estimates of receipt of mental health services vary between states and territories (58% ─89.1%) with women at a higher risk for postpartum depression less likely to receive any screening for depression [12].

A growing body of research has considered the role of social support in protecting the physical, mental and emotional wellbeing of those exposed to stress. Social support has been identified as imperative for mental wellbeing and improving postpartum outcomes especially for first-time mothers at a higher risk of psychological distress [13–17]. Social support is broadly defined as social resources (e.g., emotional, informational, instrumental) that individuals perceive to have available when needed or has been actually provided to them from their social system–both formal support (e.g., health professionals) and informal support (e.g., partners, family members, and communities) [18]. This distinction between perceived and actual support available is important as studies have found that perceived social support exerts a more substantial effect on maternal mental health and wellbeing than actual receipt of support [19, 20]. Cohen and colleagues pointed out that individuals with high levels of perceived available support tend to believe that they have the resources to cope with difficult situations and feel in control and therefore are less likely to cognitively appraise those situations as stressful compared to those with low levels of perceived available support [18].

There is substantial evidence that those with greater social support have better mental health compared to those with insufficient social support. However, most studies have used cross-sectional data that assessed mental health condition at a single point in time [21–23]. Longitudinal cohort studies provided mixed findings, with some studies identifying the association between social support and postpartum mental health while others suggest no association [24–29]. For example, a longitudinal study that followed 5,219 women over five waves (13 years period) found no protective relationship between social support- positive social interaction and postpartum depression [28]. Leahy and colleagues found that compared to professional support (e.g., midwives), informal support from family and friends was a significant predictor of maternal mental health at 6 weeks post-delivery [29].

Although previous studies improved our understanding on social support and maternal mental health, the empirical evidence on the casual effects of social support remains limited. There is still a gap in the current understanding on whether it is perceived support from relationships (formal and informal) and social contacts that affects individual’s mental wellbeing or whether it is poor mental health that affects an individual’s appraisal of social support as inadequate and which in turn worsen their mental wellbeing. The direction of simultaneity bias resulting from this association could go in either direction [30]. For example, women experiencing depression may be less likely to engage in social activities which could affect their opportunity to form or maintain relationships. The perceived lack of social support can then worsen their mental health and subsequently bias their perceived support availability as they transit to motherhood. If the reverse effect of mental health on perceived social support is assumed to be zero, this confounds the causal effect of social support and biases the estimate [47].

To our knowledge, this is the first study to examine the effect of perceived social support on maternal mental health by using a nationally representative panel data of Australian women over 17 years period. This study used fixed effects regression methods to control for observed and unobserved time-invariant heterogeneity and included dependent variable lags to account for their past mental health condition before birth (i.e., pre-pregnancy and prenatal period) which has hitherto been overlooked in previous studies. This study also extends previous research by being the first to account for the causal relationship between perceived support availability and maternal mental health.

## Materials and methods

### Data source

This study uses the Household, Income and Labour Dynamics in Australia (HILDA) survey, a longitudinal, nationally representative study of Australian households. The HILDA survey was initiated in 2001, and it collects detailed information from over 13,000 individuals within 7,000 households. The annual survey covers a range of dimensions, including social, demographics, income, health, and economic conditions obtained through a combination of a self-completion questionnaire and a face-to-face interview with trained interviewers. Although data are collected on each members of the household, interviews are only conducted with those who are above 15 years of age. The response rate for respondents who have continued in all surveys (i.e., wave-to-wave retention rate) is above 90% and 70% for new respondents who were added to the cohort in 2011 to allow a better representation of the Australian population [31].

The analytic sample consists of 3,887 births from 2,301 women who (a) reported to have given birth within the previous 12 months prior to the survey in waves 2 to 18, covering 2002 and 2018 periods and (b) reported in the subsequent year of data collection that they had a child who was under twelve months old in their residence and care. Women may be included more than once if they had given birth to more than one child during the study period. We use wave 2 (2002) as the earliest wave as data on whether respondents have given birth in the previous 12 months preceding the interview are not available in the first wave. The analyses were weighted to reflect population characteristics using responding person-level sample weights provided in the HILDA dataset. For missing data, we use the nearest observed value for the same individual for discrete variables and linear interpolation for the continuous variables. For example, missing data on ethnicity was imputed using information reported from the same individual in their earlier or later waves.

### Outcome measures

Maternal mental health was assessed using the Mental Component Summary (MCS) score derived from the Short Form 36 (SF-36). The SF-36 is a widely used self-completion 36 items survey measuring health and wellbeing and has been shown to be psychometrically sound, with good internal consistency, discriminant validity, and high reliability [32]. The SF-36 was included in every wave of the HILDA survey. Out of the 36 items in the survey, 14 are in the category of mental health and wellbeing, which can be divided into four scales that measure different aspects and components of mental health: social functioning, mental health, vitality, and role-limitation due to emotional problems. It is not designed to capture state such as anxiety and depression. This primary dependent variable, ‘MCS’ summarises the overall scores of the four scales with a range from 0 to 100, such that higher scores indicate better mental health conditions.

Although MCS has been proven to be a high-quality measure of mental health, there are concerns that it is a subjective measure and may be prone to self-reporting scale bias [32]. To address such concerns, we consider an alternative set of mental health measures in the sensitivity analyses. A similar approach has been used in other studies [33, 34]. The alternative set of mental health measures were assessed using questions from the Mental Health Inventory (MHI-5), a subscale of the self-completed Medical Outcomes Study 36-Items Short Form Health Survey developed to assess mood and anxiety disorders over the past 4-week period. Respondents reported on a 6-point Likert scale with options ranging from “1. None of the time” to “6. All the time” to three mental health related questions: (a) have you felt calm and peaceful; (b) have you felt down; (c) have you felt so down in the dumps that nothing could cheer you up. The response to each item i.e., (a) felt calm and peaceful (b) felt down (c) felt so down in the dumps were converted to an ordered categorical variable so that all items on a higher scale correspond to better mental health. This MHI-5 measure has been shown to be a good proxy for an individual’s usual state of mental wellbeing and has been widely used in the medical literature [35, 36] as well as in the health economics literature [37, 38]. Further, a continuous depression index from the SF-36 was used to assess depression severity during the first year postpartum. The response to this dependent variable, ‘Depression’ severity scale ranges from 0–100 with higher value implies better mental health.

### Perceived social support measure

In this study, our measure of social support is based on an individual’s perception of social support as this has been recognised as having a more important effect on mental health than actual social support received [18, 39]. Here, we construct measure of perceived social support based on a 10 items scale which asked respondents about their perception of social support–emotional and practical. The measure also asked about loneliness (‘I often feel very lonely’), which we would conceptualize as the converse of social support. The HILDA social support measure has been used in previous studies [40, 41]. The 10-items are rated on a seven-point Likert scale ranging from strongly disagree (1) to strongly agree (7). Five items are reverse-scored so that higher scores reflect better-perceived support. The overall social support scale is created by averaging the ten items in the scale, with lower scores representing lower social support and vice versa. The continuous social support score was coded into a 3-category summary measure representing 25^th^ (Low, score <47), 50^th^ (Medium, score 47–61) and 75^th^ (High, score >61) percentile of social support. The 3-category levels of social support were used to assess their impact on mental health outcomes contemporaneously, based on the assumption that perceived support would have an immediate effect on a scaled measure of mental health during the postpartum period.

The fixed-effect models were also adjusted for a number of confounder such as maternal age (in years), identified as Aboriginal or Torres Strait Islander (yes/no), speak a language other than English (yes/no), number of people living in the household, number of dependent children in the household, highest education level (Year 11 and below/Year 12/Certificate III or IV/Higher education), annual disposable income in the last financial year, negative life events in the previous 12 months (yes/no), labour force status (employed/Not in labour force and unemployed), partner’s age (in years) and his annual disposable income in the last financial year. These variables have been used in the literature on mental health [17, 32, 42, 43].

Summary statistics for the mental health outcomes and other individual-level characteristics are presented in Table 1. The negative life events were reported by individuals who have experienced the following in the previous 12 months preceding the interview (i) lost a close friend; (ii) lost a relative; (iii) lost a spouse or child; (iv) separated from spouse and; (v) being fired or made redundant. It is postulated that these negative events in particular ‘loss’ events, such as the death of a spouse or child are likely to affect mental wellbeing as evidenced in the psychological literature [44, 45]. For example, Kendler and colleagues found that the death of someone in an individual’s social network has the largest effect of all independent life events [45]. In the current sample, one in five women reported to have experienced at least one of the negative events in the previous 12 months. The model also included two years (wave)-based lags of mental health levels to control for prenatal and pre-pregnancy mental health. A similar approach has been done in other studies [43, 46, 47].

**Table 1:** Summary statistics.

Variables	N	Mean	Standard deviation	Minimum	Maximum
Mental component score	3,546	78.983	15.724	3	100
Depression severity	3,541	76.226	15.525	8	100
MHI-5 Subscale					
Felt calm and peaceful	3,869	3.482	0.778	1	4
Felt down	3,869	2.095	0.891	1	4
Felt down in the dumps	3,867	1.551	0.856	1	4
Social support scale	3,527	54.353	9.710	1	70
Mother’s age	3,887	30.688	5.770	16	56
Aboriginal or Torres Strait Islander	3,178	0.041	0.197	0	1
Speak a language other than English	3,887	1.874	0.332	1	2
Number of people living in household	3,887	4.004	1.189	2	17
Number of dependent children	3,887	1.851	0.996	0	8
Highest educational level	3,887	2.989	1.138	1	4
Annual disposable income in the last financial year (AUD)	3,887	31938	35,341	48	698,109
Negative events in previous 12 months					
Separated from spouse/ partner	3,887	1.001	0.280	1	2
Death of spouse or child	3,887	1.001	0.280	1	2
Death of other close relative	3,887	1.102	0.382	1	2
Death of a close friend	3,887	1.049	0.317	1	2
Fired/ made redundant by an employer	3,887	1.010	0.282	1	2
Labour force status	3,887	2.162	0.978	1	3
Partner’s age (years)	3,887	33.135	6.732	17	76
Partner’s annual disposal income in the last financial year (AUD)	3,887	60516	60,831	350	694,774

Note: negative income is not reported.

### Analytical approach

Longitudinal fixed effects regression was used for assessing the casual effects of perceived social support on postpartum mental health with continuous MCS score as the main outcome [48–50]. To avoid incidental parameters arising from large number of fixed effects [51, 52], we control for unobserved variation using year and Australian states and territories specific heterogeneity.

The regression started with an unadjusted model using individual fixed effects (Model 1). This is followed by Model 2 and Model 3 which adjusted for all confounders and using one and two years lagged value of maternal mental health respectively as it is possible that mental health during postpartum is affected by past mental health shocks. Model 4 adjusted for all confounders and assessed the effect of perceived social support on postpartum mental health using the first, second and third percentile (i.e., Low, Medium and High) of the conditional distribution. Specifically, Model 3 assessed the mean differences in mental health associated with ‘medium’ or ‘high’ social support compared with individual when they reported ‘low’ social support.

We conducted a separate regression model for the alternative set of mental health measures—Depression severity. For other mental health measures with categorical dependent variables [i.e., MHI-5 subscales–(a) felt calm, (b) felt down, and (c) felt down in the dumps], we run fixed effects ordered logit regression using the panel model as follows:

$$y_{it}^{*}=x_{it}^{\prime }\beta +\alpha_{i}+\tau_{t}+\varepsilon_{it}$$ (1)

where y* is the latent variable observed as an ordered dependent variable *y*, which takes the values 1 to 4, *x_it_* is a vector of covariates, while *β* is a vector of coefficients. *α* is the fixed effect (Australian states and territories) and *τ* is the year dummy and *ε* is the error term.

### Endogeneity

This study used an instrumental-variables (IV) approach to address endogeneity, arising from potential measurement error and simultaneity bias. Measurement error is a common issue when the derived value of a variable deviates from the true value. This issue often occurs with survey data such as HILDA when respondents provide socially desirable answers. It is also likely that relationship between poor mental health and social support is cyclical wherein both mental health and perceived support are determined simultaneously [53]. When this occurs, it is statistically challenging to isolate the independent effect of one variable (e.g., social support) on the other (e.g., mental health).

In this case, a valid IV should be used to predict social support but it should have no direct effect on health other than through social support. Based on these criteria, we employed two instrumental variables—(a) population density at state and territories level and (b) whether the respondent has been the victim of a crime (e.g., theft or housebreaking) during the last 12 months based on two-stage least squares (2SLS) approach. These variables have been used as instrument in previous studies assessing social support and health [54, 55]. Living in a higher density area is associated with greater access to services and facilities which provide opportunities for people to meet and interact compared to lower density areas with limited access to network and resources [56].

The second instrument using having been a victim of a crime is related with some degree of trust towards other people. It is not merely the lack of trust but a more pronounced suspiciousness of the motives of other people’s actions [57]. This negative experience is likely to incite individuals to draw inward due to feeling of fear and insecurity of people, results in doubts about whether they could rely on others for necessary help and support.

We constructed population density by taking annual estimated resident population relative to the area size in square kilometres for all the states and territories. Previous crime exposure was based on a crime-related item–“victim of a property crime (e.g., theft, housebreaking)” in HILDA self-completed question on “which major events have happened in their life during the past 12 months”.

All statistical analyses were performed with STATA/SE, version 16.0 (Stata Corp LP, College Station, Texas) statistical software.

## Results

Our final sample consists of 3,887 births from 2,301 women between the years 2002 (wave 2) and 2018 (wave 18). The mean age of women who have given birth in the 12 months from the pooled waves is 30.6 years (SD 5.7). Most women are non-Aboriginal or Torres Strait Islander, have completed at least Year 12 or equivalent, not in the labour force or unemployed. On average, these women have on average 1.85 children and a personal disposable income of AU$35,341 in the past financial year amongst those in employment. About one in six women (17.3%) have experienced a negative life event in the previous 12 months preceding the interview. The mean age of their partner is 33.1 years with a mean personal disposable income of AU$60,516 in the past financial year.

**Fig 1:**
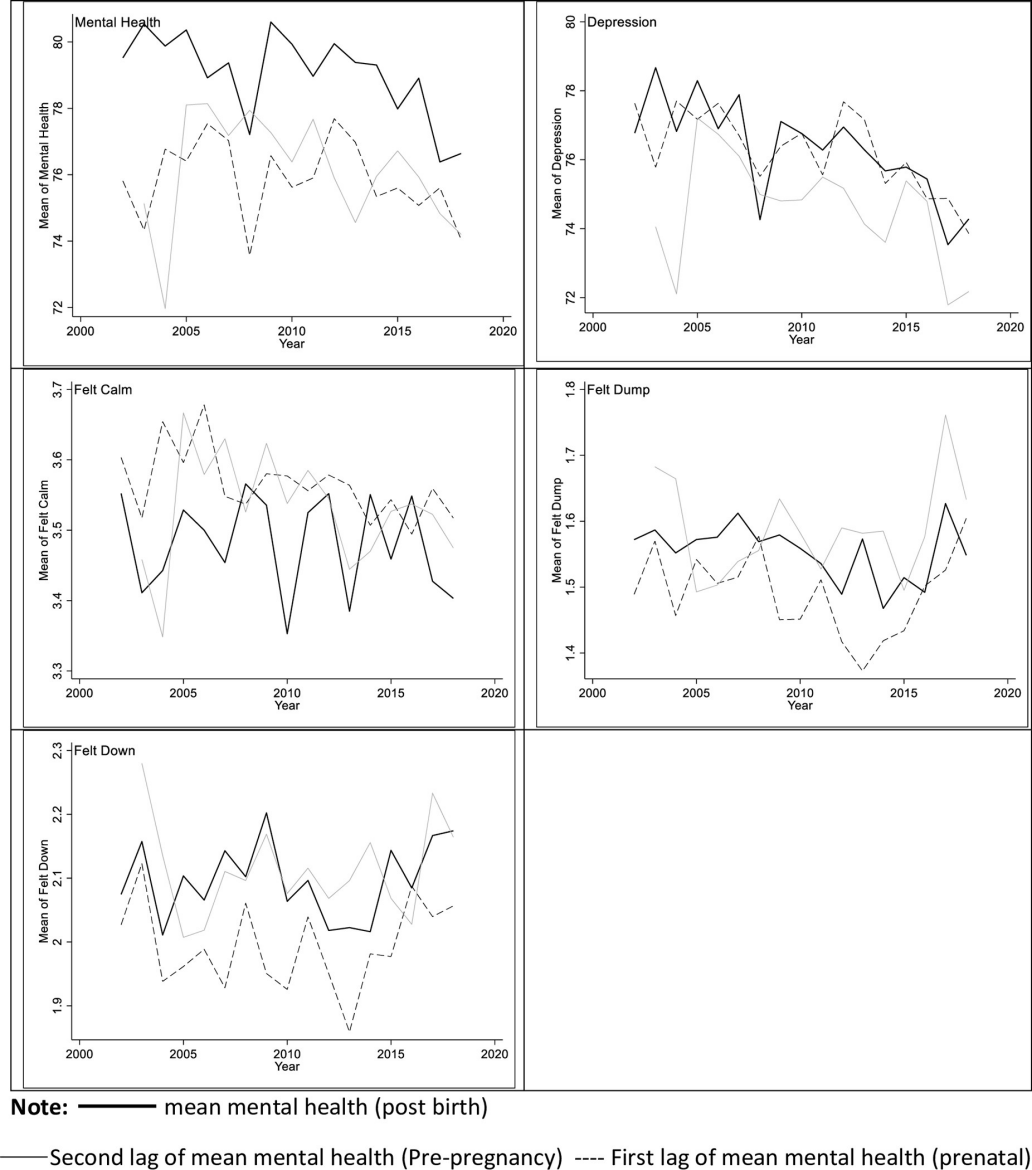
Time-varying means on past and contemporaneous mental health series and their two lags (i.e., pregnancy and pre‐pregnancy periods).

Fig 1A–1E show the mean of both current and the two-years lagged values of three mental health measures [i.e., MCS, Depression severity and three MHI-5 subscales (i.e., felt calm and peaceful, felt down, and felt down in the dump)] over the study period. The figures showed a fluctuating downward trend in the mental health levels without conditioning on other variables. The mean of the primary outcome–MCS score is 79.5 and 76.6 for wave 2 and wave 18, respectively, indicating that contemporaneous mental health during the first-year postpartum has declined. The graphs also showed a positive correlation between past mental health (i.e., pre-pregnancy and pregnancy periods) and mental health during postpartum, suggesting a considerable persistence of mental health condition. This is also shown in Table 2 (Models 2 and 3) with the significant coefficient on the one and two years lagged value of mental health levels.

**Table 2:** Mental health (Mental Component Summary) and social support.

Variables	Model 1	Model 2	Model 3	Model 4
Lag 1 of mental health (MCS)	-0.342	0.139***	0.117***	0.159***
	(0.000)	(0.000)	(0.001)	(0.000)
Lag 2 of mental health (MCS)			0.088***	
			(0.001)	
Social support scale	0.000	0.621***	0.565***	
	(0.000)	(0.001)	(0.001)	
Social support -Q2 (“Medium”)				8.515***
				(0.017)
Social support -Q3 (“High”)				12.579***
				(0.018)
Mother’s age		-0.090***	-0.275***	0.021**
		(0.009)	(0.009)	(0.008)
Mother’s age squared		0.207***	0.398***	0.058***
		(0.013)	(0.013)	(0.013)
Aboriginal or Torres Strait Islander		-1.694***	-2.617***	-2.326***
		(0.037)	(0.041)	(0.035)
Speak a language other than English		-0.460***	-0.509***	-0.546***
		(0.018)	(0.021)	(0.017)
Number of people living in household		0.644***	0.765***	0.692***
		(0.012)	(0.013)	(0.011)
Number of dependent children		-0.357***	-0.236***	-0.336***
		(0.014)	(0.016)	(0.013)
Highest education level		0.216***	0.112***	0.453***
		(0.007)	(0.008)	(0.007)
Lag of annual disposable income		0.316***	0.125***	0.399***
		(0.007)	(0.008)	(0.007)
Separated from spouse/ partner		-1.715***	-1.465***	-0.898***
		(0.030)	(0.032)	(0.025)
Death of spouse or child		0.263***	1.461***	0.120***
		(0.030)	(0.032)	(0.028)
Death of close relative		-0.810***	-0.905***	-0.843***
		(0.020)	(0.022)	(0.019)
Death of close friend		-0.648***	-0.657***	-0.865***
		(0.023)	(0.025)	(0.022)
Fired/ made redundant by an employer		2.663***	0.697***	2.815***
		(0.025)	(0.041)	(0.025)
Labour force status		0.061***	0.187***	-0.164***
		(0.015)	(0.016)	(0.014)
Partner’s age		-0.086***	-0.056***	-0.091***
		(0.002)	(0.002)	(0.001)
Partner’s annual disposal income in the last financial year		0.913***	0.804***	0.758***
		(0.005)	(0.006)	(0.005)
Individual fixed effects	Yes	No	No	No
Wave fixed effects	Yes	Yes	Yes	Yes
State and territories fixed effects	Yes	Yes	Yes	Yes
Constant	125.260	32.557***	37.737***	54.278***
	(0.000)	(0.171)	(0.190)	(0.165)
Observations	3,792,438	3,350,643	2,597,806	3,791,363

Note: Dependent variable is the Mental Component Summary score. Robust standard errors are in parentheses (clustered at the individual level). The continuous social support score was coded into a 3-category summary measure representing 25^th^ (Low, score <47), 50^th^ (Medium, score 47–61) and 75^th^ (High, score >61) percentile of social support.

*** p<0.01

** p<0.05.

Our results also showed compared to a mother with low social support, an increased to ‘medium’ support availability was associated with an 8.5-point improvement in mental health (95%CI 8.4–8.6). There was over a 12.6-point increase in postpartum mental health (95%CI (95%CI 12.5–12.7) when perceived social support increased from low to a high social support category (Model 4).

For sensitivity check, we run the fixed effect models using alternative set of mental health measures–Depression severity and three mental health items of MHI-5 (i.e., “felt calm and peaceful”, “felt so down in the dumps” and “felt down”). Similar results to the primary analysis were obtained when Depression severity was used as the dependent variable. Compared to when a new mother with low social support, an increased to ‘medium’ support was associated with a 10.2-point improvement in mental health (95%CI 10.2–10.3) and 18.0-point increase in maternal mental health (95%CI 17.9–18.1) (Table 3).

**Table 3:** Mental health (Depression severity) and social support.

Variables	Model 1	Model 2	Model 3	Model 4
Lag 1 of mental health (Depression)	0.016***	0.102***	0.080***	0.120***
	(0.001)	(0.001)	(0.001)	(0.001)
Lag 2 of mental health (Depression)			0.064***	
			(0.001)	
Social support scale	0.471***	0.767***	0.744***	
	(0.001)	(0.001)	(0.001)	
Social support -Q2 (“Medium”)				10.255***
				(0.020)
Social support -Q3 (“High”)				18.016***
				(0.023)
Mother’s age		-0.202***	-0.503***	-0.209***
		(0.010)	(0.010)	(0.010)
Mother’s age squared		0.223***	0.553***	0.186***
		(0.014)	(0.015)	(0.015)
Aboriginal or Torres Strait Islander		-3.894***	-3.852***	-4.012***
		(0.043)	(0.046)	(0.045)
Speak a language other than English		-0.975***	-1.569***	-1.477***
		(0.021)	(0.024)	(0.022)
Number of people living in household		0.751***	0.530***	0.614***
		(0.013)	(0.015)	(0.014)
Number of dependent children		-0.412***	-0.274***	-0.195***
		(0.016)	(0.017)	(0.016)
Highest education level		0.093***	0.120***	0.054***
		(0.008)	(0.009)	(0.008)
Lag of annual disposable income		0.099***	-0.244***	0.301***
		(0.008)	(0.009)	(0.008)
Separated from spouse/ partner		-1.555***	-1.151***	-1.622***
		(0.032)	(0.035)	(0.033)
Death of spouse or child		2.520***	3.126***	2.822***
		(0.033)	(0.035)	(0.034)
Death of close relative		-2.394***	-2.546***	-2.675***
		(0.023)	(0.024)	(0.024)
Death of close friend		-1.585***	-1.336***	-1.549***
		(0.025)	(0.027)	(0.026)
Fired/ made redundant by an employer		2.510***	1.402***	2.640***
		(0.028)	(0.044)	(0.029)
Labour force status		-0.663***	-0.551***	-0.577***
		(0.017)	(0.018)	(0.017)
Partner’s age		0.092***	0.120***	0.072***
		(0.002)	(0.002)	(0.002)
Partner’s annual disposal income		0.795***	0.653***	0.838***
		(0.006)	(0.006)	(0.006)
Individual fixed effects	Yes	No	No	No
Wave fixed effects	Yes	Yes	Yes	Yes
State and territories fixed effects	Yes	Yes	Yes	Yes
Constant	58.719***	23.560***	34.623***	54.745***
	(0.450)	(0.200)	(0.213)	(0.204)
Observations	3,149,719	3,045,303	2,459,626	3,059,740

Note: Dependent variable is continuous Depression severity level. Robust standard errors are in parentheses (clustered at the individual level). The continuous social support score was coded into a 3-category summary measure representing 25^th^ (Low, score <47), 50^th^ (Medium, score 47–61) and 75^th^ (High, score >61) percentile of social support.

*** p<0.01

** p<0.05.

This correlation is consistent in all three MHI-5 mental health related items. As these items are ordered variables, we employed ordered logistic model. While the results were consistent with those estimated in the main analysis (i.e., MCS score), the coefficients showed more negligible impacts of social support on maternal mental health (Table 4).

**Table 4:** Mental health (MHI-5 subscales) and social support.

Variables	Felt Calm	Felt Down	Felt Dump
Lag 1 of mental health (MHI-5)	0.141***	0.036***	0.017***
	(0.016)	(0.004)	(0.002)
Lag 2 of mental health (MHI-5)	0.188***	0.025***	0.013***
	(0.016)	(0.003)	(0.002)
Social support scale	0.007***	-0.001***	-0.001***
	(0.001)	(0.000)	(0.000)
Mother’s age	-0.010	0.002	0.001
	(0.013)	(0.003)	(0.001)
Mother’s age squared	0.013	-0.004	-0.002
	(0.020)	(0.004)	(0.002)
Aboriginal or Torres Strait Islander	-0.049	-0.005	-0.006
	(0.066)	(0.012)	(0.007)
Speak a language other than English	-0.138	0.001	-0.005
	(0.085)	(0.014)	(0.007)
Number of people living in household	-0.038	0.008**	0.003
	(0.021)	(0.004)	(0.002)
Number of dependent children	0.038	-0.003	-0.002
	(0.024)	(0.005)	(0.003)
Highest education level	-0.004	0.001	-0.002
	(0.012)	(0.002)	(0.001)
Lag of annual disposable income	0.013	-0.001	-0.000
	(0.013)	(0.003)	(0.001)
Separated from spouse/ partner	-0.002	-0.003	-0.005
	(0.039)	(0.009)	(0.004)
Death of spouse or child	0.071	0.007	0.002
	(0.040)	(0.009)	(0.005)
Death of close relative	-0.017	0.003	0.003
	(0.033)	(0.006)	(0.003)
Death of close friend	-0.073	0.010	0.004
	(0.039)	(0.007)	(0.004)
Fired/ made redundant by an employer	0.038	-0.008	-0.011**
	(0.047)	(0.010)	(0.005)
Labour force status	-0.018	0.001	-0.001
	(0.024)	(0.005)	(0.003)
Partner’s age	0.002	-0.000	0.000
	(0.003)	(0.001)	(0.000)
Partner’s annual disposal income	-0.009	-0.002	0.001
	(0.009)	(0.002)	(0.001)
Year dummy	Yes	Yes	Yes
State and territories dummy	Yes	Yes	Yes
Observations	2,033	2,031	2,023

Note: Social support scale is continuous.

*** p<0.01

** p<0.05. Fixed effects ordered logit regression.

### Endogeneity

Table 5 reported the 2SLS results that corrects for potential endogeneity of social support variable. Although the IV model showed consistent findings with former specifications, the small increment in the coefficient suggests that when social support is treated as exogenous, their effect on maternal mental health is likely underestimated (coefficient 0.635 with FE and 0.746 with IV-FE).

**Table 5:** Model with endogeneity.

Variables	Model 1	Model 2	Model 3	Model 4
Lag 1 of mental health	0.139***	0.139***	0.092***	0.092***
	(0.001)	(0.001)	(0.001)	(0.001)
Social support scale	0.635***	0.635***	0.746***	0.746***
	(0.001)	(0.001)	(0.002)	(0.002)
Mother’s age	-0.394***	-0.392***	-0.051***	-0.041***
	(0.010)	(0.010)	(0.012)	(0.012)
Mother’s age squared	0.606***	0.603***	0.008	-0.004
	(0.015)	(0.015)	(0.017)	(0.017)
Aboriginal or Torres Strait Islander	-1.796***	-1.782***	-4.180***	-4.125***
	(0.050)	(0.050)	(0.057)	(0.057)
Speak a language other than English	0.176***	0.185***	-0.832***	-0.799***
	(0.023)	(0.023)	(0.027)	(0.027)
Number of people living in household	0.692***	0.693***	0.286***	0.263***
	(0.017)	(0.017)	(0.020)	(0.020)
Number of dependent children	-0.244***	-0.300***	0.126***	-0.043*
	(0.020)	(0.021)	(0.024)	(0.024)
Highest education level	0.286***	0.278***	0.111***	0.081***
	(0.009)	(0.009)	(0.010)	(0.010)
Lag of annual disposable income	0.738***	0.748***	0.265***	0.295***
	(0.009)	(0.009)	(0.011)	(0.011)
Separated from spouse/ partner	-3.158***	-3.156***	-2.574***	-2.564***
	(0.037)	(0.037)	(0.041)	(0.041)
Death of spouse or child	0.460***	0.449***	2.658***	2.621***
	(0.033)	(0.033)	(0.037)	(0.037)
Death of close relative	-1.064***	-1.050***	-1.912***	-1.865***
	(0.025)	(0.025)	(0.028)	(0.028)
Death of close friend	-0.996***	-0.994***	-2.223***	-2.224***
	(0.029)	(0.029)	(0.033)	(0.033)
Fired/ made redundant by an employer	3.667***	3.669***	3.172***	3.172***
	(0.029)	(0.029)	(0.032)	(0.032)
Labour force status	-0.418***	-0.426***	-1.562***	-1.572***
	(0.019)	(0.019)	(0.022)	(0.022)
Partner’s age	-0.071***	-0.073***	0.129***	0.123***
	(0.002)	(0.002)	(0.002)	(0.002)
Partner’s annual disposal income	0.795***	0.792***	0.713***	0.700***
	(0.007)	(0.007)	(0.007)	(0.007)
First time mother		-0.279***		-0.925***
		(0.021)		(0.025)
Wave fixed effects	Yes	Yes	Yes	Yes
State and territories fixed effects	Yes	Yes	Yes	Yes
Constant	32.875***	33.175***	21.327***	22.426***
	(0.211)	(0.212)	(0.249)	(0.251)
Observations	2,060,153	2,060,153	1,872,087	1,872,087

Note: Social support scale is continuous. Models 2 and 4 include first time mother as co-variates.

*** p<0.01

** p<0.05. Fixed effect instrumental variables.

## Discussion

Our study shows that social support is a significant protective factor for maternal mental health. The result holds for other mental health measures (i.e., Depression severity and three MHI-5 mental health items) and across different percentile of perceived support. Our finding is similar to earlier studies that found perceived social support is important to buffer the effects of postpartum depression [58, 59].

We also found evidence that past mental health (i.e., the pre-pregnancy and pregnancy periods) is associated with postpartum mental health. This result is consistent with longitudinal studies that reported women experiencing depressive symptoms during pregnancy are at high risk of postpartum depression [27, 60, 61].

Indeed, the risk factors for postpartum depression are complex, and it is possible that there is an interplay of genetic and environmental factors. Previous studies have examined a range of individual and environmental correlates of postpartum depression [62, 63]. Depression and anxiety during pregnancy, history of psychiatric illness, being unsatisfied with marital relationships, lower socioeconomic status, and other stressful life events have been associated with an increased risk of postpartum depression [63, 64]. Other studies found maternal self-efficacy in infant care, and sufficient parenting skill are crucial in modulating the risk of postpartum depression [65, 66].

Given that poor prior mental health affect postpartum mental health, our results support the need for routine universal screening that provides a clear pathway to access additional treatment supports (e.g., mental health specialists) and equity of access to mental health care [67]. This is important especially when not all women have access to network for support such as migrant women who often have poor social network that could provide informational and instrumental support to access psychosocial care services [68]. Women who have access to family and friends may feel that their family and friends were unable to be supportive due to a lack of understanding on postpartum depression which could worsen their maternal mental health or act as a barrier to treatment [26, 69]. Previous economic analysis found maternal depression places a considerable burden on the healthcare system, and one study in 2012 estimated an annual healthcare cost of AUD443 million in Australia [70].

In the last decade, several national initiatives have been introduced including the NPDI to improve prevention and early detection of antenatal and postnatal depression. However, screening coverage for expectant mothers remains suboptimal with one in five mothers who have reported emotionally distressed during pregnancy were not being screened both antenatally and postnatally as recommended by clinical practice guidelines [71]. Of those who were screened, almost two in five perinatal Australian women (38.9%) were not comfortable with enquiry about their depression or anxiety symptoms. There were concerns that psychosocial assessment process during depression screening were causing unnecessary discomfort as women were asked to open up personal issues past trauma, domestic violence and mental health issues without evidence-based treatment pathways in place. Forder and colleagues reported that about one in five women were not being completely honest at depression screening [72].

The development of effective interventions such as provision of informational and structural resources, and the fostering of psychological support will be important to improve mental health. However more research is needed to examine how mothers perceive and use the support and the extent to which perceived social support (as evaluated by the individual) is effective. This is necessary as it is the perceived ‘supportiveness’ of others that is crucial in determining whether one would reach out to others for help and which in turn could affect their mental health outcomes. Previous studies have found many aspects of support are indicative of having someone to provide the emotional and informational support–whether it is professional or personal source of support [17, 73].

A strength of this study is the application of rigorous analytic approach including IV to a large, nationally representative cohort study, controlling for a wide array of potential confounders, prior and contemporaneous mental health and unobserved time-invariant heterogeneity. By using dependent variable lags to account for their past mental health condition before birth (i.e., pre-pregnancy and prenatal period), this study showed the persistent effect of past mental health (i.e., pre-pregnancy and pregnancy period) on contemptuous mental health (postpartum period) which has hitherto been overlooked in previous studies.

### Limitation

The study has some limitations. First the current study used a self-assessed subjective measures of perceived social support (exposure) and mental health (outcome) which could result in measurement error. But as we are primarily interested in measuring perceived social support, self-report is appropriate. Although the HILDA survey is a national, population-based study, initial response rate in the first wave was 66% which suggests that it may not be wholly representative of Australians. It is possible that a greater retention of individuals with better mental health and higher socio-economic status in the HILDA survey may introduce some bias into the results. However, loss to follow-up in consecutive HILDA waves was low with less than 10% for most waves [31]. Finally, it is also unclear to what extent our results can be generalised beyond the Australian setting given the different mental health policies introduced over the study period.

## Conclusions

Our fixed effects regression analysis of a national represented data provides strong evidence that social support is positively associated with mental health during postpartum. Past mental health affects current mental health. Women suffering from poor mental health at pre-pregnancy and during pregnancy are more likely to experience poor mental health at postpartum.

## Supporting information

S1 Fig Number of births, 2002–2018. (DOCX)

S2 Fig Number of women with first births, 2002–2018. (DOCX)
