# Supplementary material for: Perceived social support on postpartum mental health: An instrumental variable analysis
Source: PLoS One. 2022 May 5;17(5):e0265941. doi: 10.1371/journal.pone.0265941 (PMC9070871; doi:10.1371/journal.pone.0265941)
Supplement: S1 Fig — (DOCX) [file pone.0265941.s001.docx]

**Supplementary Figure 1: Number of birth, 2002- 2018**
